# Clinical spectrum and outcomes of idiopathic inflammatory myopathies in South Africans

**DOI:** 10.3389/fmed.2023.1097824

**Published:** 2023-02-13

**Authors:** Candice Birch, Mohammed Tikly, Nimmisha Govind

**Affiliations:** ^1^Department of Internal Medicine, Faculty of Health Sciences, School of Clinical Medicine, University of the Witwatersrand, Johannesburg, South Africa; ^2^Division of Rheumatology, Department of Internal Medicine, Faculty of Health Sciences, School of Clinical Medicine, University of the Witwatersrand, Johannesburg, South Africa

**Keywords:** dermatomyositis, polymyositis, cutaneous features, antibodies, corticosteroids, malignancies

## Abstract

**Background:**

Idiopathic inflammatory myopathies (IIM) are rare diseases for which there is a paucity of data in Africa. We undertook a retrospective records review of clinical and laboratory features of patients with IIM attending a tertiary service in Gauteng, South Africa.

**Materials and methods:**

Case records of patients seen between January 1990 and December 2019 and fulfilling the Bohan and Peter criteria for IIM were reviewed for demographics, clinical features, special investigations and drug therapy.

**Results:**

Of 94 patients included in the study, 65 (69.1%) had dermatomyositis (DM) and 29 (30.9%) had polymyositis (PM). Overall, the mean (SD) age at presentation and disease duration were 41.5 (13.6) and 5.9 (6.2) years, respectively. 88 (93.6%) were Black Africans. The most common cutaneous features in DM patients were Gottron’s lesions (72.3%) and abnormal cuticular overgrowth (67.7%). Dysphagia was the most common extra-muscular feature (31.9%), more so in PM than DM (*p* = 0.02). Creatine kinase, total leucocyte count and CRP were similarly higher in PM than DM patients (*p* = 0.006, 0.002, and 0.01, respectively). Anti-nuclear and anti-Jo-1 antibodies were positive in 62.2 and 20.4% of patients tested, respectively, the latter significantly more in PM than DM patients (*OR* = 5.1, *p* = 0.03) and more likely to be positive with ILD (*p* = 0.001). Corticosteroids were prescribed in all patients, 89.4% had additional immunosuppressive drugs and 6.4% required intensive/high care. Malignancies occurred in three patients, all of whom had DM. There were seven known deaths.

**Conclusion:**

The present study provides further insights into the spectrum of clinical features of IIM, especially cutaneous features of DM, anti-Jo-1 antibodies and associated ILD, in a cohort of predominantly black African patients.

## 1. Introduction

Idiopathic inflammatory myopathies (IIM) are autoimmune diseases that affect predominately skeletal muscle and skin. The estimated global prevalence of IIM is 14/100,000 (1). These diseases cause significant morbidity and mortality (2).

Polymyositis (PM) and dermatomyositis (DM) are the main variants of IIM, with differences in immunopathogenesis and clinical presentation that include the presence of characteristic skin lesions in DM. Myositis-specific antibodies (MSA) occur in about 50% of patients of which anti-Jo-1 antibodies are most common (3). Corticosteroids are first line treatment. Methotrexate and azathioprine are indicated as steroid-sparing therapy.

This study investigated the clinical, laboratory, electromyographic (EMG) and histological abnormalities, therapy and outcomes in patients with DM or PM attending a hospital in Gauteng, South Africa.

## 2. Materials and methods

A retrospective records review of patients with IIM attending a tertiary Connective Tissue Clinic in southern Gauteng, South Africa, between January 1990 and December 2019 was performed. Only patients who fulfilled the 1975 Peter and Bohan criteria for IIM (4) ≥18 years at diagnosis and had at least 3 months of follow-up data, were included. Data on patients with clinically amyopathic DM (CADM) according to the Sontheimer criteria (5), were documented separately. Patients with overlap connective tissue diseases were excluded from the analysis.

Data extracted from clinical records included demographics, clinical features, EMG, laboratory, histological findings and immunosuppressive drug therapy. Clinical findings of cutaneous features and other extra-muscular organ involvement, malignancies and comorbidities were recorded. Baseline full blood count, erythrocyte sedimentation rate (ESR), C-reactive protein (CRP), creatine kinase (CK), aldolase, aspartate transaminase (AST) and alanine transaminase (ALT), and HIV, antinuclear antibody (ANA) (by indirect immunofluorescence) and anti-Jo-1 antibody (by fluorescent enzyme immunoassay) status during the course of the disease, were also captured. Drug therapy data included use of corticosteroids, methotrexate, azathioprine, mycophenolate mofetil (MMF), cyclophosphamide, rituximab and intravenous immunoglobulins (IVIg). The study was approved by the Human Research Ethics Committee of the University of the Witwatersrand (M190565).

### 2.1. Statistical analysis

The Chi-square test or two-tailed Fisher’s exact test was applied to compare nominal variables between groups, and Student’s *T*-test or Mann–Whitney *U*-test was applied for continuous normal and non-normal distributed data, respectively. All analyses were done using TIBCO Statistica v.13.3.0 (TIBCO Software Inc., Palo Alto, CA, USA; 2017). A *p*-value < 0.05 was considered significant.

## 3. Results

Of the 138 records reviewed, 94 fulfilled the study criteria. Additionally, 8 met CADM criteria. Among the 94 patients who met Bohan and Peter criteria for IIM, most patients were black females (81.9%), with a mean age and follow-up duration of 41.5 and 5.9 years, respectively (Table 1). Median (IQR) creatine kinase (CK) for the overall cohort was 3,437 (921–7,495). Baseline muscle enzymes (CK and/or aldolase) were raised in 96.8% of patients. Subgroup analysis showed significantly higher CK and aldolase in the PM group than the DM group (*p* = 0.006 and *p* = 0.04, respectively). In those patients in whom EMG and muscle histology were performed, 87.5 and 89.6% respectively were compatible with an inflammatory myopathy.

**TABLE 1:** Demographic and clinical characteristics of 94 patients with idiopathic inflammatory myopathy.

Variable	Total (*n* = 94)	DM (*n* = 65)	PM (*n* = 29)	*P*-value
Females, *n* (%)	82 (87.2)	59 (90.8)	23 (79.3)	0.2
Black African, *n* (%)	88 (93.6)	59 (90.8)	29 (100)	0.2
Age at diagnosis, mean (SD), in years	41.5 (13.6)	41.2 (14.3)	42.2 (11.9)	0.6
Disease duration from diagnosis, mean (SD), in years	5.9 (6.2)	5.2 (5.6)	4.9 (7.1)	0.3
**Bohan and Peter criteria**				
Proximal myopathy, *n* (%)	94 (100)	65 (100)	29 (100)	1.0
Raised muscle enzymes *n* (%)	91 (96.8)	62 (95.4)	29 (100)	0.6
Creatine kinase U/l, median (IQR)*	3,437 (921–7,495)	2,354 (654–5,050)	7,180 (1,868–10,000)	0.006
Aldolase U/l, median (IQR)* (*n* = 61)	26 (14–56)	20 (12–49)	39 (26–65)	0.04
Typical electromyographic changes, *n* (%)	63/72 (87.5)	42/50 (84.0)	21/22 (95.4)	0.3
Abnormal muscle histology, *n* (%)	60/67 (89.6)	40/43 (93.0)	20/24 (83.3)	0.2
**Dermatologic features**				
Calcinosis cutis, *n* (%)		7 (10.8)	-	
Cuticular disease/ragged cuticles, *n* (%)		44 (67.7)	-	
Gottron’s lesions**, *n* (%)		47 (72.3)	-	
Heliotrope rash, *n* (%)		40 (61.5)	-	
Shawl Sign, V sign, Holster sign, *n* (%)		26 (40.0)	-	
Mechanic’s hands, *n* (%)		6 (9.2)	-	

*At presentation, **Gottron’s papules and Gottron’s sign. DM, dermatomyositis; PM, polymyositis; SD, standard deviation; IQR, interquartile range.

As shown in Table 2, dysphagia was the most common extra-muscular manifestation occurring in about a third of patients, significantly more in the PM group than the DM group (*p* = 0.03). Patients who reported dysphagia had higher baseline AST levels [median (IQR) = 262 (69–494) vs. 101 (58.5–246) compared to patients without dysphagia, *p* = 0.04] and a trend toward being more likely to be treated with IV pulse corticosteroids [9/30 vs. 8/63 without dysphagia, OR 95% CI = 2.9 (1.0–8.6), *p* = 0.05]. Raynaud’s phenomenon, inflammatory arthritis and interstitial lung disease (ILD) were observed in approximately a quarter of patients. Seven patients were clinically documented to have anti-synthetase syndrome, with specifically anti-Jo-1 antibodies. Malignancies of breast cancer (*n* = 1), lymphoma (*n* = 1), and lung cancer (*n* = 1) occurred exclusively in the DM group. None of CADM patients had any documented malignancies. Ten (10.8%) of the patients tested positive for HIV.

**TABLE 2:** Other clinical and laboratory features and outcomes in 94 patients with idiopathic inflammatory myopathy.

Variable	Total (*n* = 94)	DM (*n* = 65)	PM (*n* = 29)	*P*-value
Raynaud’s phenomenon, *n* (%)	24 (25.5)	20 (30.8)	4 (13.8)	0.1
Arthritis, *n* (%)	24 (25.5)	18 (27.7)	6 (20.7)	0.6
Dysphagia, *n* (%)	30 (31.9)	16 (24.6)	14 (48.3)	0.03 OR 95% CI = 2.9 (1.1-7.2)
Interstitial lung disease, *n* (%)	23 (24.5)	15 (23.1)	8 (27.6)	0.8
Malignancies, *n* (%)	3 (3.2)	3 (4.6)	0 (0)	0.6
**Laboratory findings**				
ANA, *n* (%)	56/90 (62.2)	44/62 (68.8)	12/28 (42.9)	0.02 OR 95% CI = 3.0 (1.2-7.3)
Anti-Ro, *n* (%)	14/54 (25.9)	10/39 (23.1)	4/13 (33.3)	0.72
Anti-Jo-1, *n* (%)	11/54 (20.4)	4/36 (11.1)	7/18 (38.9)	0.03 OR 95% CI = 5.1 (1.2-20.8)
Anemia**, *n* (%)*	24/89 (27.0)	18/64 (28.1)	6/20 (30.0)	0.8
Total WCC X10^9^, median (IQR)*	7.5 (5.5–10.0)	7.0 (5.0–9.0)	9.2 (7.3–12.0)	0.002
Platelet count X10^9^, median (IQR)*	319 (252–397)	303 (248–393)	359 (296–404)	0.1
CRP (mg/L), median (IQR)*	9.5 (4.6–42.5)	8.0 (4.0–24.0)	37.2 (7.0–80.1)	0.01
ESR (mm/hr), median (IQR)*	27 (15–51)	25 (15–42)	29 (17–62)	0.3
ALT (U/L), median (IQR)*	92 (37–222)	83 (32–173)	157 (61–288)	0.04
AST (U/L), median (IQR)*	114 (59–322)	105 (58–283)	259 (98–404)	0.06

*At presentation, DM, dermatomyositis; PM, polymyositis; OR, odd’s ratio; CI, confidence interval; ANA, antinuclear antibody; CRP, C-reactive protein; ESR, erythrocyte sedimentation rate; WCC, white cell count; AST, aspartate transaminase; ALT, alanine aminotransferase; ICU, intensive care unit; HCA, high care area. **Anemia in females *Hb* < 12 g/dl, anemia in males *Hb* < 13 g/dl.

Anemia, leucocytosis and thrombocytosis at presentation were noted in 27, 25, and 13.5% of patients, respectively. Baseline median white cell count (*p* = 0.002), CRP (*p* = 0.01), and ALT (*p* = 0.04) were higher in the PM group than the DM group. Most patients had raised AST (85.5%) and ALT (71.1%) levels >40 U/l.

The ANA test was positive in 62.2% of patients, more in the DM group than the PM group (*p* = 0.02). Anti-Jo-1 antibody positivity was lower in the DM group (*p* = 0.03). Moreover, none of the patients who tested anti-Jo-1 antibody positive had a heliotrope rash [0/21 vs. 11/33 without heliotrope, OR 95% CI = 0.09 (0.01–0.73), *p* = 0.003]. In contrast, patients with ILD were more likely to test positive for anti-Jo-1 antibodies [8/18 vs. 2/35 without ILD, OR 95% CI = 13.2 (2.4–72.5), *p* = 0.001]. There was no significant association between anti-Jo-1 antibodies and arthritis (*p* = 0.15) and Raynaud’s phenomenon (*p* = 0.45). Patients with ILD had a lower AST [median (IQR) = 77.5 (52–125) vs. 178 (67–385.5) in patients without ILD, *p* = 0.03] and a lower CK at baseline [median (IQR) = 1,105 (463–4,539) vs. 3,648 (1,178–8,088) in patients without ILD, *p* = 0.05]. A quarter of patients tested positive for anti-Ro antibodies and those who were anti-Jo-1 antibody positive were more likely to be anti-Ro positive (*OR* = 21, 95% CI = 1.83–240.52, *p* = 0.001).

All patients were treated with oral prednisone and 18.1% required intravenous methylprednisolone (Table 3). Most, 84 of 94 (89.4%), were treated with at least one or more immunosuppressive drug. Methotrexate was the most commonly prescribed immunosuppressant (72.3%), followed by azathioprine (42.6%), mycophenolate mofetil (MMF) (24.4%) and cyclophosphamide (16%). Patients with ILD were more likely to have been treated with MMF [10/23 vs. 13/70 patients with no ILD, OR 95% CI = 3.4 (1.2–9.4), *p* = 0.02] and cyclophosphamide [13/23 vs. 2/70 patients with no ILD, OR 95% CI = 44.2 (8.7–225.6), *p* < 0.0001]. Rituximab and intravenous immunoglobulin therapy were prescribed in 17 and 6.4% of patients, respectively. Chloroquine was prescribed mostly in DM patients for cutaneous disease (53.8%) and in a minority (10.3%) of PM patients for arthritis.

**TABLE 3:** Immunosuppressive therapy and outcomes in 94 patients with idiopathic inflammatory myopathy.

Variable	Total (*n* = 94)	DM (*n* = 65)	PM (*n* = 29)	*P*-value
Prednisone	94 (100)	65 (100)	29 (100)	1.0
IV prednisone	17 (18.1)	13 (20.0)	4 (13.8)	0.6
Any steroid-sparing agent	84/94 (89.4)	58/65 (89.2)	26/29 (89.7)	1.0
MTX	28 (29.8)	19 (29.2)	9 (31.0)	1.0
CYC	15 (16.0)	10 (15.4)	5 (17.2)	1.0
MMF	2 (2.1)	1 (1.5)	1 (3.4)	0.5
AZA	8 (8.5)	8 (12.3)	0 (0)	0.2
RTX	16 (17.0)	8 (12.3)	8 (27.6)	0.08
IVIG	6 (6.4)	4 (6.2)	2 (6.9)	1.0
Chloroquine	38 (40.4)	35 (53.8)	3 (10.3)	0.0001
**Outcome**				
Hospital admissions	67 (71.3)	44 (67.7)	23 (79.3)	0.3
ICU/HCA admissions	6 (6.4)	3 (4.6)	3 (10.3)	0.3
Demised	7 (7.5)	5 (7.7)	2 (6.9)	1.0
Lost to follow up	39 (41.2)	24 (36.9)	15 (51.7)	0.3

DM, dermatomyositis; PM, polymyositis; IV, intravenous; MTX, methotrexate; CYC, cyclophosphamide; MMF, mycophenolate mofetil; AZA, azathioprine; RTX, rituximab; IVIG, intravenous immunoglobulin; ICU, intensive care unit; HCA, high care area.

Overall, 67 (71.3%) patients required hospital admission, with six (6.4%) admitted to ICU or high care for respiratory support. Seven (7.4%) deaths were recorded, five patients with DM and two with PM, and 39 (41.2%) patients were lost to follow-up overall. Known causes of death were one each of metastatic lung cancer, infection, and cor pulmonale with congestive cardiac failure. The causes of death in the remaining four patients were not known.

The eight patients with CADM had a similar spectrum and frequency of cutaneous features to DM with respect to Gottron’s lesions (72.3 vs. 87.5%), nail-fold cuticular overgrowth (67.7 vs. 62.5%), heliotrope rash (72.3 vs. 62.5%), shawl/V sign/holster sign (40.1 vs. 37.5%), and calcinosis cutis (11.8 vs. 12.5%).

## 4. Discussion

In this retrospective study of mainly indigent blacks with IIM attending a tertiary rheumatology center, most patients had DM in whom Gottron’s lesions, the pathognomonic cutaneous feature of the disease (6), and abnormal nail-fold cuticular overgrowth were the commonest features. Patients with PM had more active and severe disease at presentation as evidenced by a higher frequency of dysphagia and higher muscle enzymes, white cell counts and CRP compared to the DM patients.

Overall, our clinical findings, admission rates and drug therapies are largely like those reported previously (2, 7–9). Consistent with studies in other populations most patients were female. Peak age of presentation in the fifth decade is similar to studies from other developing countries where most patients present in the third to fifth decades (2, 9), in contrast to patients from North America (10) and Europe who generally present later in the sixth decade (11).

The predominance of DM patients largely reflects the referral system at our institution where PM patients are mostly referred to Neurology. However, previous studies have suggested UV light exposure in tropical and subtropical regions may explain the higher prevalence of DM in Asia and Africa (8). On the other hand, PM had more severe disease. Dysphagia, which has been shown previously to be associated with weakness of neck and respiratory muscles (12), was common in PM patients. Like in the above study, our patients with dysphagia were more likely to have pulse IV corticosteroid therapy. Moreover, the CK, aldolase, AST and ALT were higher in the PM patients, in keeping with the findings in a US study (13). In the only other recent sub-Saharan African study of 104 patients with IIM, including those with overlap syndromes, Chinniah et al. found no difference in baseline CK levels between patients with DM and PM and found dysphagia to be more common patients with DM (7). The small number of HIV + patients precluded meaningful statistical comparisons. Almost three quarters of patients in the present study had one or more hospital admission, like that reported in an Egyptian cohort (14).

The overall ANA positivity in just under two-thirds of all patients, but more common in DM than PM patients, is not too dissimilar from previous reports where a positive ANA has been found in 24–60% of DM and 16–40% of PM (15). The Jo-1 antibody test, the only myositis specific antibody test recorded in the study, was present in one-fifth of patients in whom the test was done, more so in patients with PM than DM and in patients with ILD, as reported previously in several studies (3). The relevance of anti-Jo-1 autoantibodies in patients with definite polymyositis and ILD is being increasingly recognized as common and potentially serious complication of IIM. In the present study, almost a quarter of the patients were documented to have ILD, similar to the 30% reported IIM in the EuroMyositis registry and occurred most frequently in association with the anti-synthetase syndrome (11).

Malignancies were documented in only three patients (3.2%) in the present study and exclusively in patients with DM similar to the 4.8% in the Chinniah study. Previous studies have consistently shown malignancies to be more common in patients with DM than PM, although overall frequency of malignancies is highly variable in different populations and as high as 45 and 27% of DM and PM patients, respectively in an Israeli study (16). These ethno-geographical differences are thought to be due a combination of factors including referral bias, differences in screening for malignancies, and genetic and environmental factors (15).

The drugs used to treat the IIM in the present study are comparable to the current international standard of care with methotrexate and azathioprine being the most commonly prescribed corticosteroid sparing agents (11, 17). Chloroquine has only been evaluated in observational studies for the management of skin in DM and was reported to be effective in 40–75% of patients (18).

Limitations of the study include the retrospective nature of the work, particularly with respect to missing data, inconsistencies in documenting clinical features and screening for malignancies, and a substantial proportion of patients lost to follow-up. Moreover, there have been major advances in diagnosis of extra-articular features during the study period, especially with respect to detecting ILD. Newer myositis specific antibodies are not yet readily available. There has also been a change in drug therapy in recent years, particularly the greater availability of rituximab and MMF.

Notwithstanding the limitations, the present study provides further insights into the spectrum of clinical features and differences between patients with DM and PM of predominantly black African extraction. Prospective studies are necessary to better understand the impact of IIM on health-related quality of life and life expectancy in relation to clinical subsets and myositis-specific antibodies.

## Data availability statement

The raw data supporting the conclusions of this article will be made available by the authors, without undue reservation.

## Ethics statement

The studies involving human participants were reviewed and approved by the Human Research Ethics Committee of the University of the Witwatersrand, Johannesburg (M190565). Written informed consent for participation was not required for this study in accordance with the national legislation and the institutional requirements.

## Author contributions

MT and NG contributed to the concept, design, definition of intellectual content, manuscript editing, manuscript review, involved in statistical analysis, and manuscript preparation. CB contributed to the literature search, data acquisition, data analysis, statistical analysis, and manuscript preparation. All authors contributed to the article and approved the submitted version.
